# Hawthorne Effect: More Than Just Telephones

**DOI:** 10.31486/toj.22.5031

**Published:** 2022

**Authors:** Lavern E. Nossaman, Bobby D. Nossaman

**Affiliations:** ^1^ Western Electric, Shreveport, LA; ^2^ Department of Anesthesiology and Perioperative Medicine, Ochsner Clinic Foundation, New Orleans, LA; ^3^ The University of Queensland Medical School, Ochsner Clinical School, New Orleans, LA

## INTRODUCTION

Translating results from published clinical trials to everyday clinical practice is not a straightforward process. Observational trials generally are conducted at the beginning of investigations to determine the effectiveness of a therapeutic intervention. However, observational trials suffer from *confounding by indication*, when decisions for a particular treatment are based upon clinical presentation.^1,2^ Randomized controlled trials (RCTs) are then developed to minimize confounding by indication, as randomization ensures that both measured and unmeasured confounders are, on average, balanced between the groups of interest.^2,3^ Yet RCTs are not representative of the patient population as they suffer from design restraints, restrictive enrollment criteria, patient participation, and clinical disease variability, and can also lack external validity.^4-7^ One additional confounder of RCTs is *observational bias*, when the patient and/or caregiver modifies responses to care because they are aware of the study conditions. The modification of responses as a result of being observed, or the *Hawthorne effect*, has its origins in the telephone equipment manufacturing industry.^1,7-9^

## HISTORY OF THE HAWTHORNE WORKS

Telecommunication equipment, including telephones, for the American Telephone & Telegraph Company (AT&T, “Ma Bell”) was manufactured at the Western Electric Company Hawthorne Plant in Illinois from the 1920s to the mid-1960s and then transferred to the Shreveport plant in Louisiana. To improve manufacturing productivity, research was conducted in a series of observational trials to measure changes in productivity following experimental alterations in the workplace environment. Following initial experiments on workplace lighting, a special assembly workroom was established to allow production behavior to be carefully monitored following additional changes in the environmental conditions. Implementation of rest and lunch breaks, different work hour shifts, and choosing one's coworkers initially improved productivity, but once observation ceased, productivity returned to near-normal levels.^10^ These changes in observed productivity were later termed the Hawthorne effect.^9^ This effect has been studied in other disciplines such as social psychology, industrial and organizational psychology, management theory, industrial sociology, psychiatry, and now in medicine.^10^

## HAWTHORNE EFFECT IN MEDICINE

The Hawthorne effect has been reported in perioperative studies. Nakayama et al,^11^ Teernstra et al,^12^ and Kwaan et al^13^ had unexpected improvements in their control groups when compared to similar control groups from earlier pilot or published studies.^14^ These authors credited the differences in the latter studies to provider surveillance.^11-13^ Improvements in interventional studies have also been reported because of the Hawthorne effect.^15-18^ Hence, the Hawthorne effect can occur when either the patients and/or health care workers are aware of the study conditions, which poses difficulty when generalizing the results to clinical practice. Eventually, the clinical effectiveness of any new medication needs re-examination under real-world, non-Hawthorne effect conditions.^1,7-9^

## CONFOUNDERS

Although RCTs should evenly distribute known and unknown factors between groups,^7^ in clinical practice, therapies are not randomized, thereby introducing confounding by indication. In a report by Benson and Hartz, who examined treatment outcomes in 19 diverse medical and surgical treatments compared with both RCT and non-RCT methods, the outcome effect sizes were similar, with only 2 of 19 analyses dissimilar to the point where differences in treatment effects fell outside of the 95% confidence intervals.^19^ McKee et al reported that neither method consistently gives larger estimates of treatment effect.^20^

Eventually, when proposing interventions from clinical trials during everyday clinical care, patient, family, and social preferences, as well as physician preferences, will influence therapeutic decisions.^20^ The introduction of a novel therapeutic then must rise above this background noise of confounders to be an effective signal in demonstrating improvement in patient care.

## INITIAL STUDIES WITH SUGAMMADEX

Neuromuscular blocking agents are frequently used to facilitate endotracheal intubation and improve surgical working conditions during general anesthesia.^21-24^ Reversal of neuromuscular blockade is frequently required to facilitate return of airway muscle function, including adequate ventilation. This reversal has been achieved through the use of acetylcholinesterase inhibitors, most commonly neostigmine, since the 1950s.^25,26^ However, in 2015, the FDA approved the use of the novel cyclodextrin, sugammadex, in reversing rocuronium- or vecuronium-induced neuromuscular blockade.^27^ Based upon the speed of sugammadex in reversing neuromuscular blockade when compared to neostigmine^28,29^ and with initial comparative studies reporting improved operating room discharge and postanesthesia care unit recovery times,^30,31^ editorials^32,33^ proposed that the routine use of this modified gamma-cyclodextrin could provide additional time savings and improve perioperative productivity.

**Figure 1.**
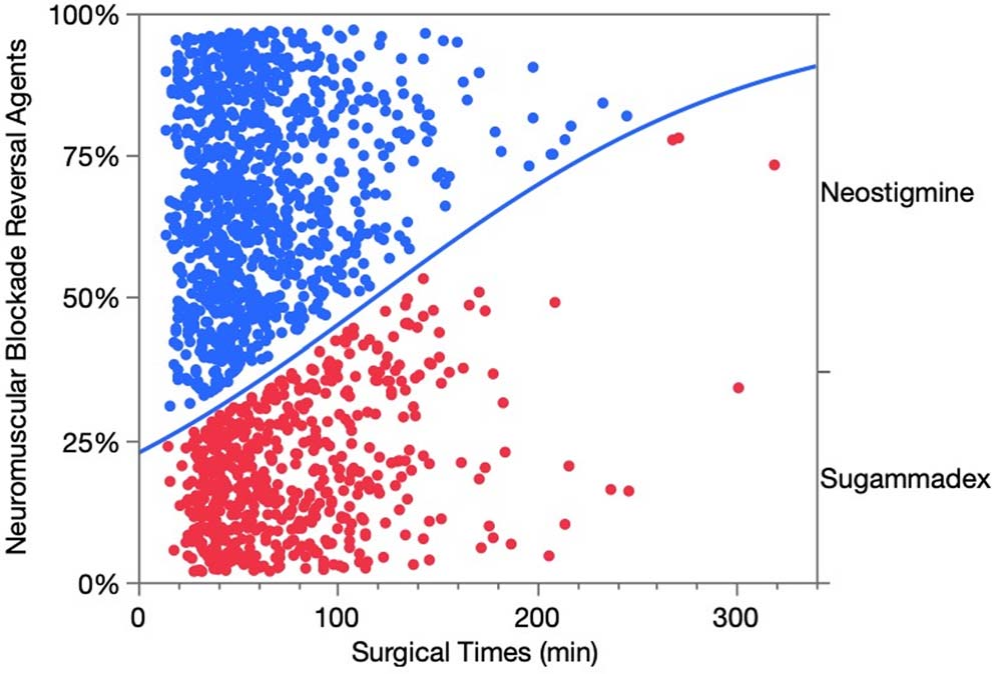
**Association of surgical times in minutes (min) and neuromuscular blocking reversal agent—sugammadex in red and neostigmine in blue—chi-square=120, *P*<0.0001. *P* values <0.005 are statistically significant.^36^** (For readers of the print publication, a color version of this figure is available online at https://doi.org/10.31486/toj.22.5031.)

**Figure 2.**
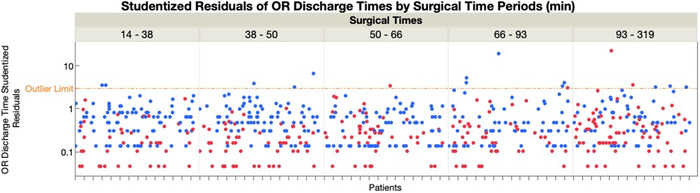
**Overlay plot of studentized residuals (sugammadex in red and neostigmine in blue) of operating room (OR) discharge times by increasing surgical time periods in minutes (min). Studentized residuals^41^ are displayed in logarithmic scale to improve clarity of the graph. The outlier limit for studentized residuals was set to 3 in this analysis.** (For readers of the print publication, a color version of this figure is available online at https://doi.org/10.31486/toj.22.5031.)

A 2022 study by Moss et al examined the role of the neuromuscular blocking reversal agents on operating room times.^34^ Although sugammadex was associated with shorter operating room times of ∼12.5 minutes, the time savings with sugammadex were largely associated with shorter surgical times.^34^ We conducted a post hoc analysis of reversal agents by surgical times^35^ and found that as surgical times for laparoscopic cholecystectomy increased, a higher percentage of sugammadex was used for reversal of rocuronium-induced neuromuscular blockade (Figure 1). Lower median and interquartile range [IQR] surgical times for neostigmine (53 [IQR 39-75] minutes) were observed when compared to sugammadex (64 [IQR 42-99] minutes) surgical times (chi-square=120, *P*<0.0001).^36^ These post hoc results suggest that the duration of the surgical procedure played a role in the selection of the neuromuscular reversal agent. If sugammadex use was delegated to longer, probably unexpected, laparoscopic cholecystectomy surgical times, then the quicker reversal effect reported for sugammadex, when compared to neostigmine,^28,37-40^ should translate into less variance or spread in operating room discharge times, including the number of outliers. We parceled surgical times into 5 groups, with the studentized residuals^41^ of the operating room discharge times plotted against the type of neuromuscular blocking reversal agent (Figure 2). Log-linear variance analysis of the residuals, a manufacturing production regression technique,^42,43^ is shown in the Table.

**Table.** Log-Linear Variance Analysis of Operating Room Discharge Time Residuals by Neuromuscular Reversal Agent in 1,611 Patients Undergoing Laparoscopic Cholecystectomy

Neuromuscular Blockade Reversal Agent	Variance Parameter Estimate	95% CI	SE	Chi-Square	*P* Value
Sugammadex	1.1	1.03-1.2	0.04	881	<0.0001
Residual	108	101-116	4.0	735	<0.0001

Notes: Neostigmine is the active comparative control. *P* values <0.005 are statistically significant.^36^

The overlay plot in Figure 2 suggests a pattern of smaller values of studentized sugammadex residuals when compared to the neostigmine studentized residual values. More studentized neostigmine outliers were observed than studentized sugammadex outliers (values >3), with greater numbers observed in the higher surgical time periods. The log-linear analysis of the residuals shows that the variance of sugammadex was statistically different than that of the active control neostigmine (Table). These analyses support the hypothesis^34^ that in cases with longer surgical times, sugammadex may allow for faster completion of surgery as the number of studentized sugammadex outliers was less (Figure 2). Nevertheless, in the study by Lee, Ahsan, Chae, et al,^35^ the authors explored the perioperative efficiency of this gamma-cyclodextrin for improving operating room discharge and postanesthesia care unit recovery times without favorable results.

In summary, confounding occurs during general clinical practice, and clinical investigation needs to address Hawthorne effect conditions.^9^ When translating results from published clinical trials, novel therapeutics need to rise above this background noise to demonstrate improvements in patient care.
